# Sex difference in the prognostic role of body composition parameters in Taiwanese patients undergoing transcatheter aortic valve implantation

**DOI:** 10.1186/s12872-020-01569-z

**Published:** 2020-06-10

**Authors:** Hsiao-Huang Chang, Po-Lin Chen, Hsin-Bang Leu, I-Ming Chen, Nai-Yuan Wu, Ying-Hwa Chen

**Affiliations:** 1 Division of Cardiovascular Surgery, Department of Surgery, Taipei Veterans General Hospital, Taipei, Taiwan; 2 Department of Surgery, School of Medicine, Taipei Medical University, Taipei, Taiwan; 3 Department of Biomedical Sciences and Engineering, National Central University, Taoyuan, Taiwan; 4 Department of Medicine, School of Medicine, National Yang-Ming University, Taipei, Taiwan; 5 Division of Cardiology, Department of Internal Medicine, Taipei Veterans General Hospital, No.201, Sec. 2, Shipai Rd., Beitou District, Taipei, 11217 Taiwan; 6 Institute of Biomedical Informatics, National Yang-Ming University, Taipei, Taiwan

**Keywords:** Aortic stenosis, Body mass index, Body surface area, Lean body mass, Mortality, Transcatheter aortic valve implantation

## Abstract

**Background:**

Evidence on association between body composition and outcomes of transcatheter aortic valve implantation (TAVI) is limited for Asian patients. This study investigated the prognostic role of body composition parameters in Taiwanese patients undergoing TAVI.

**Materials and methods:**

Data of consecutive patients undergoing TAVI for severe aortic stenosis between May 1, 2010 and August 31, 2019 were prospectively collected in this observational study. The association between body composition parameters (body mass index [BMI], body surface area [BSA], lean body mass [LBM], and LBM index) and cumulative mortality was analyzed using Cox proportional hazard regression model.

**Results:**

A total of 221 patients (mean age 81.4 years), including 125 (56.6%) males, were included with median follow-up duration of 23.8 months. In males, multivariate analysis revealed that higher BMI (*P* = 0.035), BMI ≥ 20 kg/m^2^ (*P* = 0.026), and higher LBM index (*P* = 0.023) significantly predicted lower overall all-cause cumulative mortality. In females, none of the body composition parameters was significantly associated with all-cause cumulative mortality. Paradoxical association between BMI and estimated all-cause cumulative mortality was only significant among male patients.

**Conclusion:**

In Taiwanese TAVI patients, the prognostic effects of BMI and LBM index on cumulative mortality were only observed in males, not in females. Sex differences must be considered when stratifying risk among patients undergoing TAVI.

## Background

Clinical medicine relies on measures of body composition, or habitus, to guide treatment decisions and to predict clinical outcomes. Commonly used measures include body mass index (BMI), body surface area (BSA), and measurement of fat, bone, and lean masses. BMI is the most frequently used measure of body habitus, but may not always account for true body fat mass [1]. Women with higher BMI have higher fat mass and lean mass according to dual-energy x-ray absorptiometry than those with lower BMI, and men with higher BMI also have higher fat mass while lean mass is similar to that in men with lower BMI [2]. BSA is reported to be a stronger predictor of one-year survival than BMI in patients with chronic heart failure; survival is longer in these patients when the overall size of the body is greater, regardless of height correction [1]. Lean body mass (LBM) independently predicts all-cause mortality in patients with coronary heart disease [3]. Overweight and obese status are associated with significant risk for cardiovascular, metabolic and musculoskeletal diseases and, many cancers as well [4].

Nevertheless, while obesity is recognized as an independent risk factor for cardiovascular morbidity and mortality in the general population, [5] epidemiologic studies have observed the phenomenon of obesity paradox [6–9]. The obesity paradox refers to the inverse relationship between body mass and mortality in chronic heart failure patients with different degrees of disease severity [6]. That is, obesity appears to provide protective effects within the general population, including in patients with heart failure, [7] atrial fibrillation, [8] and patients undergoing percutaneous coronary intervention [9]. Several studies also have reported the phenomenon of obesity paradox in patients undergoing transcatheter aortic valve implantation (TAVI) [10, 11]. In a review and meta-analysis of 13 studies, lower BMI was associated with a poorer prognosis after TAVI; obese patients had similar outcomes as normal weight patients but had significantly better long-term survival, and underweight patients had a higher incidence of major vascular complications and serious bleeding [10]. Another meta-analysis of 16 studies with 12,330 patients also reported that higher BMI was significantly associated with lower 30-day mortality and better long-term all-cause mortality in patients undergoing TAVI [11]. TAVI patients are notably at higher risk of morbidity and mortality with conventional aortic valve replacement surgery, and the less invasive TAVI has been shown to reduce morbidity and mortality substantially in many patients, though not in all [12]. Therefore, it has become especially important to identify patients at higher risk of poor outcomes of TAVI and to understand the specific associated prognostic factors.

While the above-cited studies have emphasized the paradoxical link between higher BMI and lower risk of TAVI-related mortality, evidence is lacking on possible association between body composition and prognostic factors for TAVI outcomes in Asian populations, which are known to have distinctly different body composition and body size than Western populations [13]. The meta-analysis by Sannino et al. cited above included no studies of Asian populations, [10] and Asian patients only accounted for 10.2% of the included patients in the meta-analysis by Lv et al. [11] In the recent literature, the prognostic role of body composition parameters for Asian individuals undergoing TAVI appears to have been investigated in only two published studies [14, 15]. Therefore, the present study aimed to investigate the prognostic role of body composition parameters in a cohort of Taiwanese patients undergoing TAVI.

## Methods

### Study design and subjects

This observational study is a retrospective analysis of a prospectively collected database. The database records a cohort of consecutive patients who underwent TAVI between May 1, 2010 and August 31, 2019 at Taipei Veterans General Hospital. Patients who were diagnosed with severe aortic stenosis by clinical, echocardiographic and hemodynamic examinations were evaluated by our cardiovascular team for suitability and eligibility for TAVI [16]. Demographic, anthropometric, and clinical data of those patients deemed suitable for TAVI treatment were then recorded in the database. All TAVI procedures were performed under general or local anesthesia. Access site decisions were made by the cardiovascular team under consideration of diameter and tortuosity of femoral and iliac arteries, the aorta, and individual patient factors. All patients were followed until September 30, 2019 or until death.

### Ethical considerations

All included patients provided signed informed consent to receive TAVI treatment and for their data to be recorded for later evaluation. The protocol for this observational study was approved by the Institutional Review Board of Taipei Veterans General Hospital (No. 2017–09-002CC).

### Data collection

Data collection included demographic information, medical history, clinical data, laboratory results, and follow-up data. Clinical outcomes and complications were recorded according to the Valve Academic Research Consortium-2 consensus (VARC-2), including all-cause cumulative mortality, cardiovascular cumulative mortality, stroke or transient ischemic attack, life-threatening bleeding, acute kidney injury, coronary artery obstruction, myocardial infarction, major vascular complications, new permanent pacemaker implantation, and new onset cardiac conduction disturbance [17].

Anthropometric data, including body weight and height of all patients, were collected prospectively before TAVI treatment. Body composition parameters analyzed in this study included BMI, BSA, LBM, and LBM index. BMI was calculated as the body weight in kilograms divided by the square of the body height in meters. Because the mean body height was only 150 cm, we also calculated New BMI: 1.3 × weight [kg]/height [m]^2.5 [18]. The classification of BMI followed the recommendation of World Health Organization (WHO): normal weight (BMI < 25 kg/m^2^), overweight (25 ≤ BMI < 30 kg/m^2^), and obesity (BMI ≥ 30 kg/m^2^) [19]. The BMI cutoff of 20 kg/m^2^ was evaluated because the VARC-2 provides BMI < 20 kg/m^2^ as one of the criteria for frailty [17]. BSA was calculated by the Mosteller formula [20]. LBM was calculated by the James formula as follows: in females, LBM = 1.07 × weight [kg] − 148 × (weight [kg]/height [cm])^2^; in males, LBM = 1.1 × weight [kg] − 128 × (weight [kg]/height [cm])^2^ [21]. LBM index was calculated as LBM divided by the square of body height in meters. Estimated glomerular filtration rate (eGFR) was calculated using the Cockcroft-Gault formula.

### Statistical analysis

Data are presented as count (percentage) for categorical variables and as mean and standard deviation (SD) for continuous variables. Pearson’s chi-squared test and two-sample t-test were used for categorical variables and continuous variables, respectively, to compare outcomes between BMI groups. A propensity score based on baseline characteristics for each subject was calculated using logistic regression model. Variables in the model included age, BMI, hypertension, diabetes, hyperlipidemia, coronary artery disease, peripheral vascular disease, cerebrovascular accident, pulmonary disease, prior myocardial infarction, prior coronary artery bypass grafting, prior percutaneous coronary intervention, pre-existing pacemaker, New York Heart Association Functional Classification class III-IV, logistic score of the European System for Cardiac Operative Risk Evaluation (EuroSCORE), eGFR, atrial fibrillation, left ventricle ejection fraction, mean pressure gradient, pulmonary artery pressure, moderate or severe mitral regurgitation, and moderate or severe aortic regurgitation. Mortality rate and event rate was estimated by life table method and compared with adjustment for propensity scores. Multivariate Cox proportional hazard regression analyses were performed to evaluate the independent effect of each body composition parameter on overall and one-year all-cause cumulative mortality, respectively. Variables with *P* <  0.1 in univariate models included age, history of diabetes, history of coronary artery disease, history of peripheral vascular disease, history of cerebrovascular disease, prior coronary artery bypass grafting, prior percutaneous coronary intervention, presence of atrial fibrillation at baseline, left ventricle ejection fraction, logistic EuroSCORE, and estimated glomerular filtration rate. To avoid collinearity among covariates, only the presence of atrial fibrillation at baseline and logistic EuroSCORE were taken as covariates in the multivariate models [22]. Results of Cox proportional hazard regression analyses were presented as adjusted hazard ratios (HR) with 95% confidence interval (CI). BMI was calculated as a continuous variable as well as a categorical variable in each model. A two-tailed *P*-value of < 0.05 indicated statistical significance. All statistical analyses were performed using IBM SPSS Statistics for Windows, Version 24.0 (Armonk, NY, USA).

## Results

A total of 221 patients with mean age 81.4 years (SD = 8.4) among whom 125 (56.6%) were males were included. Median length of follow-up was 23.8 (interquartile range = 9.7–46.0) months. Table 1 presents patients’ baseline demographic, anthropometric, and clinical characteristics by gender. Although female and male patients had similar age (*P* = 0.626), BMI (*P* = 0.556), and proportion of BMI < 20 kg/m^2^ (*P* = 0.672), female patients had significantly smaller BSA (< 0.001), smaller LBM (< 0.001), smaller LBM index (< 0.001), and larger New BMI (*P* = 0.011) than male patients did. Proportions of history of coronary artery disease, percutaneous coronary intervention, and peripheral vascular disease were greater in female patients than in male patients. Additionally, female patients had significantly higher LV ejection fraction (*P* = 0.026), lesser proportion of PA pressure > 60 mmHg (*P* = 0.027), greater proportion of moderate or severe MR (*P* = 0.025), smaller aortic valve area (*P* <  0.001), smaller LV mass (*P* <  0.001), and greater proportion of valve size 23 and 25/26 (*P* <  0.001) than male patients (Tables 1, 2).

**Table 1:** Demographic characteristics and baseline clinical information

Variables	All (*n* = 221)	Female (*n* = 125)	Male (*n* = 96)	P
Age (year)*	81.4 (8.4)	81.2 (8.0)	81.7 (8.9)	0.626
Body height (cm)*	156.9 (9.5)	150.5 (6.5)	165.2 (5.7)	< 0.001
Body weight (kg)*	60.2 (12.8)	55.7 (11.2)	66.2 (12.3)	< 0.001
BMI (kg/m^2^)*	24.4 (4.2)	24.5 (4.4)	24.2 (3.9)	0.556
BMI ≥ 20 kg/m^2^	189 (85.5)	108 (86.4)	81 (84.4)	0.672
Normal weight (BMI < 25 kg/m^2^)	126 (57.0)	68 (54.4)	58 (60.4)	0.614
Overweight (25 kg/m^2^ ≤ BMI < 30 kg/m^2^)	78 (35.3)	46 (36.8)	32 (33.3)	
Obesity (BMI ≥ 30 kg/m^2^)	17 (7.7)	11 (8.8)	6 (6.3)	
New BMI (kg/m^2^)*	25.4 (4.5)	26.0 (4.8)	24.5 (3.9)	0.011
New BMI ≥ 20 kg/m^2^	192 (86.9)	111 (88.8)	81 (84.4)	0.422
Normal weight (New BMI < 25 kg/m^2^)	106 (48.0)	53 (55.2)	53 (42.4)	0.015
Overweight (25 kg/m^2^ ≤ New BMI < 30 kg/m^2^)	92 (41.6)	53 (42.4)	39 (40.6)	
Obesity (New BMI ≥ 30 kg/m^2^)	23 (10.4)	19 (15.2)	4 (4.2)	
Body surface area (m^2^)*	1.6 (0.2)	1.5 (0.2)	1.7 (0.2)	< 0.001
Lean body mass (kg)*	44.4 (8.4)	38.7 (4.5)	51.7 (6.3)	< 0.001
Lean body mass index (kg/m^2^)*	17.9 (1.8)	17.1 (1.4)	18.9 (1.8)	< 0.001
Logistic EuroSCORE (%)*	18 (15.0)	18.5 (16.1)	17.3 (13.5)	0.554
Hypertension	161 (72.9)	89 (71.2)	72 (75.0)	0.529
Diabetes	83 (37.6)	43 (34.4)	40 (41.7)	0.269
Hyperlipidemia	99 (44.8)	52 (41.6)	47 (49.0)	0.276
NYHA class III-IV	144 (65.2)	81 (64.8)	63 (65.6)	0.898
Coronary artery disease	93 (42.1)	39 (31.2)	54 (56.3)	< 0.001
Prior myocardial infarction	14 (6.3)	8 (6.4)	6 (6.3)	0.964
Prior CABG	10 (4.5)	6 (4.8)	4 (4.2)	0.822
Prior PCI	75 (33.9)	28 (22.4)	47 (49.0)	< 0.001
Peripheral vascular disease	59 (26.7)	25 (20.0)	34 (35.4)	0.010
Cerebrovascular accident	51 (23.2)	24 (19.2)	27 (28.4)	0.108
Pulmonary disease	48 (21.7)	23 (18.4)	25 (26.0)	0.172
Pre-existing pacemaker	5 (2.3)	2 (1.6)	3 (3.1)	0.655
Atrial fibrillation	47 (21.3)	29 (23.2)	18 (18.8)	0.423
Bicuspid aortic valve	26 (11.8)	14 (11.2)	12 (12.5)	0.766
Serum creatinine (mg/dl)*	1.8 (2.1)	1.6 (1.9)	2 (2.3)	0.145
eGFR (ml/min)*	40.8 (22.1)	38.7 (20.4)	43.6 (23.9)	0.101
eGFR < 60 ml/min	182 (82.4)	105 (84.0)	77 (80.2)	0.464
Dialysis	17 (7.7)	8 (6.4)	9 (9.4)	0.411
Echocardiographic findings				
LV ejection fraction (%)*	55.2 (10.2)	56.5 (9.4)	53.4 (10.9)	0.026
Aortic valve area (cm^2^)*	0.7 (0.2)	0.6 (0.2)	0.7 (0.2)	< 0.001
Mean pressure gradient (mmHg)*	44 (18.5)	45.1 (19.6)	42.5 (16.9)	0.292
Peak pressure gradient (mmHg)*	71.6 (29.3)	73.3 (30.6)	69.5 (27.5)	0.336
PA pressure > 60 mmHg	187 (86.6)	101 (82.1)	86 (92.5)	0.027
MR, moderate or severe	40 (18.1)	29 (23.2)	11 (11.5)	0.025
AR, moderate or severe	35 (15.8)	19 (15.2)	16 (16.7)	0.767
LV mass (g)*	224.7 (72.2)	207.2 (65.5)	246.3 (74.7)	< 0.001

Data were presented as count (percentage) or mean (standard deviation)*

Abbreviation: *BMI* body mass index, *EuroSCORE* European System for Cardiac Operative Risk Evaluation, *NYHA* New York Heart Association Functional Classification, *CABG* coronary artery bypass grafting, *PCI* percutaneous coronary intervention, *eGFR* estimated glomerular filtration rate, *LV* left ventricle, *PA* pulmonary artery, *MR* mitral regurgitation, *AR* aortic regurgitation

**Table 2:** Procedural data

Variables	All (*n* = 221)	Female (*n* = 125)	Male (*n* = 96)	P
Access				0.318
Transfemoral	197 (89.1)	115 (92.0)	82 (85.4)	
Transapical	9 (4.1)	3 (2.4)	6 (6.3)	
Subclavian	4 (1.8)	1 (0.8)	3 (3.1)	
Transcarotid	5 (2.3)	2 (1.6)	3 (3.1)	
Direct aortic	4 (1.8)	2 (1.6)	2 (2.1)	
Abdominal aorta	2 (0.9)	2 (1.6)	0 (0)	
Valve				0.749
CoreValve	117 (52.9)	62 (49.6)	55 (57.3)	
Evolut R	72 (32.6)	45 (36.0)	27 (28.1)	
Sapien	10 (4.5)	5 (4.0)	5 (5.2)	
Sapien-XT	15 (6.8)	9 (7.2)	6 (6.3)	
Lotus/other	7 (3.2)	4 (3.2)	3 (3.1)	
Valve size (mm)				< 0.001
23	27 (12.2)	23 (18.4)	4 (4.2)	
25/26	88 (39.8)	65 (52.0)	23 (24.0)	
29	82 (37.1)	34 (27.2)	48 (50.0)	
31/34	24 (10.9)	3 (2.4)	21 (21.9)	
Local anesthesia	130 (58.8)	75 (60.0)	55 (57.3)	0.685
Contrast volume (ml)*	108.5 (60.2)	112.3 (67.4)	103.4 (48.8)	0.288

Data were presented as count (percentage) or mean (standard deviation)*

Included patients had a median survival of 82.7 (95% CI = 45.2–120.3) months. All-cause death, overall, one-year, and 30-day cumulative mortality were 56.5, 15.5, and 4.1%, respectively. Cardiovascular death, overall, one-year, and 30-day cumulative mortality were 28.1, 6.1, and 2.7%, respectively. After adjusted with propensity score, no significant difference between female and male patients in terms of all-cause, cardiovascular cumulative mortality, and event rates of 30-day VARC2 defined outcomes (Table 3).

**Table 3:** Event rates of clinical outcomes

Variables	All (*n* = 221)	Female (*n* = 125)	Male (*n* = 96)	P^a^
Overall cumulative mortality				
All-cause	64 (56.5)	35 (44.3)	29 (63.7)	0.704
Cardiovascular	22 (28.1)	14 (25.2)	8 (28.3)	0.561
One-year cumulative mortality				
All-cause	32 (15.5)	20 (17.2)	12 (13.3)	0.601
Cardiovascular	12 (6.1)	7 (6.4)	5 (5.7)	0.762
30-day cumulative mortality				
All-cause	10 (4.1)	5 (4.0)	5 (5.2)	0.939
Cardiovascular	6 (2.7)	3 (2.4)	3 (3.1)	0.781
30-day VARC-2 defined outcomes				
Stroke or transient ischemic attack	4 (2.0)	2 (1.7)	2 (2.3)	0.997
Life threatening bleeding	7 (3.4)	2 (1.7)	5 (5.6)	0.212
Acute kidney injury, stage 2 or 3	15 (7.1)	6 (5.0)	9 (9.9)	0.751
Coronary artery obstruction	2 (1.0)	1 (0.9)	1 (1.1)	0.805
Myocardial infarction	4 (2.0)	2 (1.7)	2 (2.3)	0.387
Major vascular complications	5 (2.4)	0 (0)	5 (5.6)	0.953
New pacemaker implantation	8 (3.9)	3 (2.5)	5 (5.6)	0.142
Moderate to severe paravalvular leakage	5 (2.4)	3 (2.5)	2 (2.3)	0.825
Valve-related dysfunction requiring repeat procedure^b^	1 (0.5)	1 (0.9)	0 (0)	0.978

Data were presented as number of event (event rate). Event rate was estimated by life table method

Abbreviation: *VARC* Valve Academic Research Consortium

^a^Comparison was performed with adjustment for propensity scores

^b^Repeat procedure refers to balloon aortic valvuloplasty, transcatheter aortic valve implantation, or surgical aortic valve replacement

**Fig. 1:**
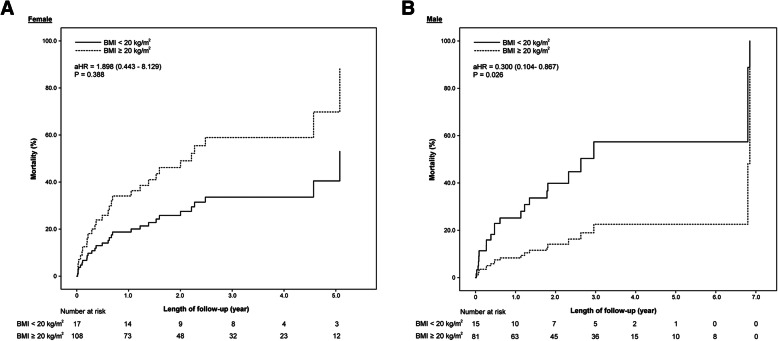
Overall all-cause mortality stratified by body mass index of 20 kg/m^2^ for female patients (**a**) and for male patients (**b**)

**Fig. 2:**
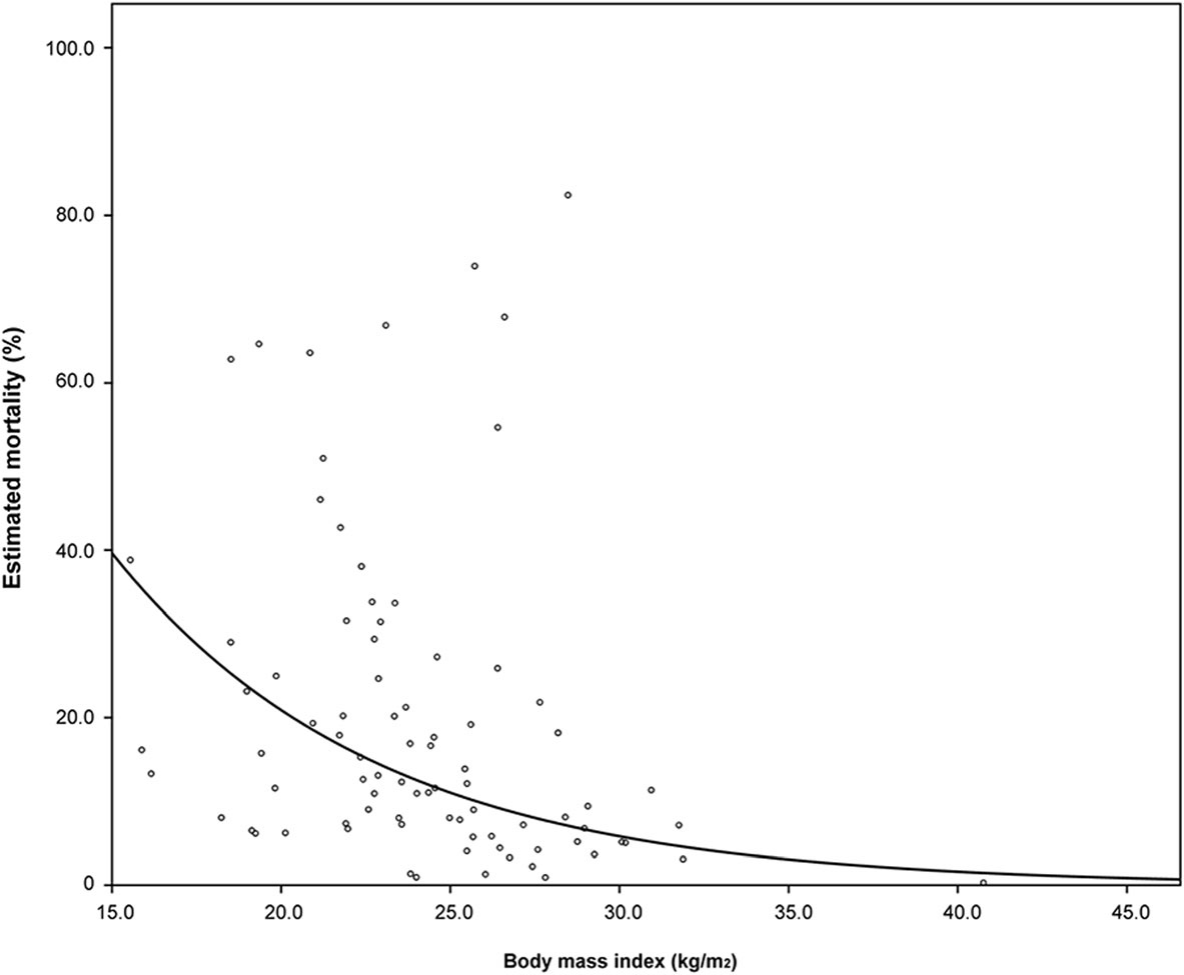
Association between body mass index and estimated all-cause mortality for male patients

For male patients, higher BMI (adjusted HR = 0.859, 95% CI = 0.746–0.989, *P* = 0.035), BMI ≥ 20 kg/m^2^ (adjusted HR = 0.300, 95% CI = 0.104–0.867, *P* = 0.026), and higher LBM index (adjusted HR = 0.734, 95% CI = 0.562–0.957, *P* = 0.023) were significantly associated with lower overall all-cause cumulative mortality, but BSA and LBM were not. Overweight patients had significant lower risk than normal weight patients did (adjusted HR = 0.156, 95% CI = 0.040–0.605, *P* = 0.007). In females, none of the body composition parameters was significantly associated with overall all-cause cumulative mortality. A similar trend was noted for one-year all-cause cumulative mortality (Table 4, Fig. 1). The paradox relationship between BMI and estimated all-cause cumulative mortality was only significant among male patients (Fig. 2). The multivariate results based on New BMI were similar to those based on current BMI (Supplementary Table 1).

**Table 4:** Multivariate analysis for overall and one-year all-cause cumulative mortality

	All		Female		Male	
Variables	Adjusted HR (95% CI)	P	Adjusted HR (95% CI)	P	Adjusted HR (95% CI)	P
Overall all-cause cumulative mortality						
BMI (per 1 kg/m^2^ increase)	1.000 (0.923–1.083)	0.988	1.066 (0.966–1.177)	0.205	0.859 (0.746–0.989)	0.035
BSA (per 1 m^2^ increase)	1.298 (0.298–5.657)	0.728	1.917 (0.170–21.629)	0.599	0.331 (0.017–6.601)	0.469
Lean body mass (per 1 kg increase)	1.094 (0.552–2.169)	0.761	1.012 (0.930–1.100)	0.790	0.972 (0.898–1.052)	0.485
Lean body mass index (per 1 kg/m^2^ increase)	0.985 (0.838–1.159)	0.859	1.192 (0.861–1.650)	0.290	0.734 (0.562–0.957)	0.023
Categorical BMI						
BMI ≥ 20 vs. < 20 kg/m^2^	0.755 (0.334–1.708)	0.500	1.898 (0.443–8.129)	0.388	0.300 (0.104–0.867)	0.026
Categorical BMI						
Overweight vs. normal weight^a^	0.692 (0.365–1.309)	0.258	1.145 (0.507–2.583)	0.745	0.156 (0.040–0.605)	0.007
Obesity vs. normal weight^a^	1.822 (0.616–5.390)	0.278	3.176 (0.834–12.094)	0.090	0.682 (0.087–5.345)	0.716
One-year all-cause cumulative mortality						
BMI (per 1 kg/m^2^ increase)	0.760 (0.889–1.090)	0.984	1.058 (0.943–1.187)	0.336	0.812 (0.676–0.976)	0.026
BSA (per 1 m^2^ increase)	0.314 (0.046–2.163)	0.239	0.615 (0.027–14.098)	0.761	0.026 (0.001–1.408)	0.073
Lean body mass (per 1 kg increase)	0.971 (0.927–1.016)	0.204	0.959 (0.860–1.069)	0.452	0.906 (0.817–1.005)	0.062
Lean body mass index (per 1 kg/m^2^ increase)	0.895 (0.725–1.104)	0.300	1.125 (0.753–1.682)	0.564	0.660 (0.473–0.922)	0.015
Categorical BMI						
BMI ≥ 20 vs. < 20 kg/m^2^	0.497 (0.199–1.237)	0.133	1.023 (0.230–4.5560	0.977	0.211 (0.058–0.774)	0.019
Categorical BMI						
Overweight vs. normal weight^a^	0.490 (0.197–1.217)	0.124	0.849 (0.280–2.574)	0.772	0.052 (0.005 0.054)	0.013
Obesity vs. normal weight^a^	2.765 (0.879–8.696)	0.082	3.744 (0.895–15.668)	0.071	1.181 (0.131–10.636)	0.882

Abbreviation: *BMI* body mass index, *BSA* body surface area, *HR* hazard ratio

^a^Normal weight: BMI < 25 kg/m^2^; overweight: 25 kg/m^2^ ≤ BMI < 30 kg/m^2^; obesity: BMI ≥ 30 kg/m^2^

## Discussion

To the best of our knowledge, the present study is the first to evaluate the prognostic value of BSA and LBM index for Asian TAVI patients. Multivariate analysis of over two-hundred patients in Taiwan who underwent TAVI showed that higher BMI, BMI ≥ 20 kg/m^2^, and higher LBM index were significantly associated with lower overall all-cause cumulative mortality and one-year all-cause cumulative mortality in male patients only, not in female patients. In females, none of the body composition parameters was significantly associated with overall all-cause cumulative mortality. Paradoxical association between BMI and estimated all-cause cumulative mortality was only significant among male patients.

Results of the present study are consistent with results from the OCEAN-TAVI registry in Japan, which revealed that BMI < 20 kg/m^2^ was associated with higher all-cause mortality for male patients, but not for female patients [14]. Results from the Asian TAVR registry revealed that higher BMI was significantly associated with lower all-cause mortality [15]. However, results of the present study, in which BMIs were higher than those in the Asian TAVR registry, found that a significant association between BMI and TAVI outcomes was only observed in male patients. Because the Asian TAVR registry did not provide results for both females and males, we cannot explain the inconsistent findings, except that the present study included fewer patients. As concluded by other authors, although body composition measures are important prognostically, BMI may be less useful as an indicator of prognosis than other, more powerful measures of habitus [1].

Regarding BSA, the present study found no associations between BSA results and overall and one-year all-cause cumulative mortality in both female and male TAVI patients. In a study of Western patients, the BSA results of Arsalan et al. were comparable to those of the present study, showing that continuous BSA was not significantly associated with 30-day mortality and one-year survival [23].

Results from the OCEAN-TAVI registry of all-Asian patients also revealed that LBM was an independent predictor of all-cause mortality in both male and female patients; i.e., low LBM had a higher incidence of all-cause death after TAVI, regardless of gender [14]. This suggests that high-risk can be identified better using LBM than conventional BMI in TAVI candidates. However, in the present study higher LBM indices were significantly associated with lower all-cause cumulative mortality in male patients only, not in female patients. The James formula, and not dual-energy x-ray absorptiometry, was used to calculate LBM in both our study and the OCEAN-TAVI study, but the inconsistent findings between the studies may possibly be explained by having fewer patients in the present study, making it more difficult to achieve statistical significance given the same effect size. Patients in the present study were also taller and had an overall greater LBM than those in the OCEAN-TAVI registry, suggesting that the LBM index may be a more suitable and sensitive indicator than LBM for stratifying risk of TAVI patients.

The present study observed sex differences in prognostic effect of body composition parameters for TAVI outcomes, which also have been noted in many previous reports. Among patients undergoing coronary artery bypass grafting, underweight as an independent risk factor for early mortality was found only in men [24]. Similarly, a meta-analysis including 16 studies of 385,925 diabetes patients revealed that overweight was associated with lower risk of all-cause mortality only in males and the risk of mortality in females did not differ between BMI subgroups [25]. In a study conducted for Japanese population, the body shape index in men, which is a calculation based on body height, weight and waist circumference, significantly increased the risk for all-cause mortality after adjusting for other known risk factors, but was not significantly associated with all-cause mortality in women [26]. In addition, among Korean heart failure patients, obesity was independently associated with lower one-year mortality rates in men, but not in women [27]. All of the reports suggest sex differences in the obesity paradox.

Sex difference in clinical outcomes after TAVI patients have reported in numerous studies. Most studies observed a similar 30-day mortality between female and male patients, but results regarding long-term mortality were not consistent across studies.e Some studies reported that female patients had more favorable survival outcomes at long-term follow-up, [28, 29] but others reported that female and male patients had similar long-term mortality [30, 31]. Our study showed that all of the 30-day, one-year, overall all-cause cumulative mortalities were similar between female and male patients after adjusting for propensity scores. The non-comparable results might be due to the different demographic characteristics and baseline comorbidities among study populations.

In several studies for Western populations, higher risk of vascular complications and life-threatening bleeding in female TAVI patients have been reported [31–33]. One explanation is that female patients have smaller vessels which may be more vulnerable to mechanical injury resulting in more bleeding events [34, 35]. But in studies for Asian populations, both the present study and the OCEAN-TAVI registry did not observe the sex difference in vascular complications and life-threatening bleeding [14]. Possible reason for the inconsistent findings may be the unique anatomical characteristics of Asian populations. As reported by Watanabe et al., Japanese patients had smaller short- and long-axis annulus diameter, sinus of Valsalva, and sinotubular junction than European patients, whether in males or females [36]. In addition to smaller BMI and BSA, Asian populations have smaller aortic valve complex and iliofemoral arteries than Western populations [35–37]. While race difference also plays an important role in these features which may theoretically increase the risk of vascular complications, life-threatening bleeding, and coronary occlusion, the effect of sex difference might be reduced by that of race difference.

This study has several limitations, including the use of observational study design, for which potential observational bias and reporting bias could not be completely avoided. In addition, the sample size in this study was not enough to perform further subgroup analysis such as stratifying by sex and BMI, as performed in previous studies [14]. Only baseline parameters were evaluated and temporal values over time were not considered. Therefore, patients with a small somatotype and those with progression to cachexia could not be distinguished. Unintentional weight loss was significantly associated with poor outcomes at 6-month and one-year follow-up, whereas BMI was not, as also shown in other studies [12]. This result suggests that changes in body habitus and their effects on TAVI outcomes need further study. Further, only 21 (14.5%) patients with BMI < 20 kg/m^2^ were included in the present study. Because the association between abdominal obesity and risk of mortality tends to be stronger in patients with lower BMI, we suggest that future studies should include waist circumference and waist-to-hip ratio in addition to BMI, especially for underweight patients, as noted previously among cancer patients [38]. In addition, only the effects of continuous LBM and LBM index were analyzed because no consensus exists for the cutoff of LBM and LBM index for Asian patients, so no categorization could be done for these parameters. Finally, all patients in this study were Taiwanese, treated in a single center, and had larger BMI and LBM than those in the Asian TAVR registry and the OCEAN-TAVI registry, illustrating that Asian populations have diverse characteristics. This study is not representative of Asian populations, but only Taiwanese population. Direct comparison of the results of this study with the Asian TAVR registry and the OCEAN-TAVI registry may therefore not be appropriate. Further multi-country and multi-center studies in Asia are necessary to explore the effect of sex difference in Asian TAVI patients.

## Conclusion

In Taiwanese TAVI patients, BMI ≥ 20 kg/m^2^, higher BMI and higher LBM index are significantly associated with lower overall and one-year all-cause cumulative mortality in male patients only, not in female patients. Sex differences must be considered when stratifying risk among patients who are being evaluated for TAVI. The effect of changes in body habitus on TAVI outcomes also deserves further investigation.

## Supplementary information

**Additional file 1: Table S1.** Multivariate analysis based on New BMI for overall and one-year all-cause cumulative mortality.

## Data Availability

The datasets used and/or analyzed during the current study are available from the corresponding author on reasonable request.

## Abbreviations

BMI: Body mass index
BSA: Body surface area
LBM: Lean body mass
TAVI: Transcatheter aortic valve implantation
VARC-2: Valve academic research Consortium-2
eGFR: Estimated glomerular filtration rate
SD: Standard deviation
EuroSCORE: European system for cardiac operative risk evaluation
HR: Hazard ratios
CI: Confidence interval
The Asian TAVR registry: The Asian Transcatheter aortic valve replacement registry
The OCEAN-TAVI registry: The optimized transCathEter vAlvular intervention- TAVI registry
