# Supplementary material for: Sex difference in the prognostic role of body composition parameters in Taiwanese patients undergoing transcatheter aortic valve implantation
Source: BMC Cardiovasc Disord. 2020 Jun 10;20:283. doi: 10.1186/s12872-020-01569-z (PMC7288684; doi:10.1186/s12872-020-01569-z)
Supplement: Supplementary file 1 — Additional file 1: Table S1. Multivariate analysis based on New BMI for overall and one-year all-cause cumulative mortality. [file 12872_2020_1569_MOESM1_ESM.docx]

**SUPPLEMENTARY MATERIALS**

Supplementary table 1. Multivariate analysis based on New BMI for overall and one-year all-cause cumulative mortality.

|  | All | | Female | | Male | |
| --- | --- | --- | --- | --- | --- | --- |
| Variables | Adjusted HR (95% CI) | P | Adjusted HR (95% CI) | P | Adjusted HR (95% CI) | P |
| Overall all-cause cumulative mortality | | | | | | |
| New BMI (per 1 kg/m^2^ increase) | 0.999 (0.925-1.079) | 0.978 | 1.069 (0.974-1.173) | 0.162 | 0.850 (0.740-0.977) | 0.022 |
| New BMI ≥ 20 vs. < 20 kg/m^2^ | 0.621 (0.275-1.404) | 0.253 | 1.386 (0.325-5.918) | 0.659 | 0.300 (0.104-0.867) | 0.026 |
| Overweight vs. normal weight^*^ | 0.641 (0.348-1.180) | 0.153 | 1.128 (0.514-2.478) | 0.763 | 0.146 (0.040-0.536) | 0.004 |
| Obesity vs. normal weight^*^ | 1.143 (0.385-3.390) | 0.809 | 1.418 (0.362-5.551) | 0.616 | 0.845 (0.109-6.549) | 0.872 |
| One-year all-cause cumulative mortality | | | | | | |
| New BMI (per 1 kg/m^2^ increase) | 0.998 (0.906-1.099) | 0.962 | 1.059 (0.948-1.182) | 0.309 | 0.818 (0.684-0.979) | 0.028 |
| New BMI ≥ 20 vs. < 20 kg/m^2^ | 0.482 (0.193-1.206) | 0.119 | 0.912 (0.203-4.104) | 0.904 | 0.211 (0.058-0.774) | 0.019 |
| Overweight vs. normal weight^*^ | 0.368 (0.149-0.908) | 0.030 | 0.562 (0.185-1.710) | 0.310 | 0.052 (0.005-0.521) | 0.012 |
| Obesity vs. normal weight^*^ | 1.859 (0.591-5.851) | 0.289 | 1.699 (0.394-7.335) | 0.478 | 1.407 (0.155-12.768) | 0.762 |

Abbreviation: BMI, body mass index; BSA, body surface area; HR, hazard ratio.

* Normal weight: New BMI < 25 kg/m^2^; overweight: 25 kg/m^2^ ≤ New BMI < 30 kg/m^2^; obesity: New BMI ≥ 30 kg/m^2^.
